# Recent Trends on IoT Systems for Traffic Monitoring and for Autonomous and Connected Vehicles

**DOI:** 10.3390/s21051648

**Published:** 2021-02-27

**Authors:** Sergio Saponara, Stefano Giordano, Riccardo Mariani

**Affiliations:** 1 DII (Dipartimento di Ingegneria della Informazione), University of Pisa, via G. Caruso 16, 56122 Pisa, Italy; stefano.giordano@unipi.it; 2 NVIDIA, Santa Clara, CA 95051, USA; rmariani@nvidia.com

## Abstract

**Internet of Things, Electronics, Embedded systems, Cyber Physical Systems, Autonomous vehicles, Connected vehicles.**

## 1. Introduction

This Editorial analyzes the manuscripts accepted, after a careful peer-reviewed process, for the special issue “IoT Sensing Systems for Traffic Monitoring and for Automated and Connected Vehicles” of the Sensors MDPI journal. The special issue has been co-organized by Professors Sergio Saponara and Stefano Giordano, both from the University of Pisa, Italy, and Riccardo Mariani, Vice President, Industry Safety at NVIDIA, USA.

As reported in Section 2 of this Editorial, the 11 selected papers give an overview of the trends in research and development activities about autonomous and connected vehicles and particularly the following: traffic density detection and prediction using closed circuit video systems and connected and autonomous probes as sensors, plus artificial intelligence (AI) techniques; advanced security schemes for over the air update of automotive software in the Internet-of-Vehicle (IoV) scenario; vehicle detection using satellite images; accurate vehicle positioning and quality of service of communication in IoV applications; Vehicle to Everything (V2X) beam alignment; multi-camera vehicle tracking using edge computing; and lightweight on-board solutions for real-time weather prediction to assist in optimal journey planning.

## 2. Recent Trends IoT Systems for Traffic Monitoring and for Autonomous and Connected Vehicles

The special issue is characterized by 11 original research papers [1,2,3,4,5,6,7,8,9,10,11].

The first paper [1], “Towards an End-to-End Framework of CCTV-Based Urban Traffic Volume Detection and Prediction”, is written by M. V. Peppa, T. Komar, Wen Xiao, P. James, C. Robson, Jin Xing, and S. Barr from the University of Newcastle, UK; and the University of Melbourne, Australia.

The paper is about near real-time urban traffic analysis and prediction for effective intelligent transport systems. A framework to support decision making in local traffic bureaus using largely available IoT sensors, especially CCTV (Closed Circuit TeleVision) systems, is yet to be developed. To this aim, this work presents an end-to-end urban traffic volume detection and prediction framework using CCTV images. The framework incorporates a novel CNN (Convolutional Neural Network) to generate vehicle counts and quantify traffic conditions. The paper investigates the performance of a statistical-based model, a random forest (RF) machine learning, and a deep learning long short-term memory (LSTM) model to predict traffic volume 30 min in the future. Tests with varying traffic conditions under different lengths of past time series are used to train the prediction models. RF and LSTM provided the most accurate predictions, with RF being faster than LSTM. The developed framework has been successfully applied to fill data gaps under adverse weather conditions when data are missing. It can be potentially implemented in near real time at any CCTV location and integrated into an online visualization platform.

The second paper [2], “Performance Evaluation of Attribute-Based Encryption in Automotive Embedded Platform for Secure Software Over-The-Air Update”, is written by M. La Manna, L. Treccozzi, P. Perazzo, S. Saponara, and G. Dini from the University of Pisa, Italy.

As developed in the framework of the European Processor Initiative, this paper shows that it is possible to improve security for Over The Air (OTA) update functionalities in an automotive scenario through the use of a cryptographic scheme, called “Attribute-Based-Encryption” (ABE), which grants confidentiality to the software/firmware update done remotely. The paper demonstrates that the overhead of the ABE integration in terms of computation time and its storage is negligible with respect to the other overheads that are introduced by the OTA process, also proving that security can be enhanced with a minimum cost. The paper reports experimental results of an implementation of the proposed ABE OTA technique on an automotive-oriented HW/SW platform equipped with a Zynq UltraScale+ MPSoC chip that is representative of the computing capability of real automotive Electronic Control Units (ECUs).

The third paper [3], “An Indoor Robust Localization Algorithm Based on Data Association Technique”, is written by Long Cheng, Yong Wang, Mingkun Xue, and Yangyang Bi. The authors are from Northeastern University, China and SANY Group, China.

The paper addresses the problem of non-line-of-sight (NLOS) transmission that reduces positioning accuracy in indoor positioning. Anchor nodes are divided into several groups, and the position information of the target node for each group is obtained through the maximum likelihood estimation (MLE). By identifying the NLOS method, a part of the position estimates polluted by NLOS transmission was discarded. For the position estimates that passed the hypothesis testing, a corresponding poly-probability matrix was established, and the probability of each position estimate from line-of-sight (LOS) and NLOS was calculated. The position of the target was obtained by combining the probability with the position estimate. In addition, the paper also considers the case where there is no continuous position estimation through hypothesis testing and through the NLOS tracking method to avoid positioning errors. Reported simulation and experimental results show that the algorithm proposed has higher positioning accuracy and higher robustness than other algorithms.

The fourth paper [4], “Vehicle Detection in Overhead Satellite Images Using a One-Stage Object Detection Model”, is authored by D. G. Stuparu, R.-I. Ciobanu, and C. Dobre from University Politehnica of Bucharest and the National Institute for Research and Development in Informatics of Bucharest, Romania.

To improve the traffic in large cities and to avoid congestion, advanced methods of detecting and predicting vehicle behavior are needed. Such methods require complex information regarding the number of vehicles on the roads, their positions, directions, etc. One way to obtain this information is by analyzing overhead images collected by satellites or drones and extracting information from them through intelligent machine learning models. To this aim, paper [4] presents a one-stage object detection model for finding vehicles in satellite images using the RetinaNet architecture and the Cars Overhead With Context dataset. The results obtained by the proposed model show that this work has a good vehicle detection accuracy and a low detection time.

The fifth paper [5] deals with resource allocation in IoV applications and is titled “Design Optimization of Resource Allocation in OFDMA-Based Cognitive Radio-Enabled Internet of Vehicles (IoVs)”. The paper is written by Eze J., Eze E., Zhang S., and Liu E. from the University of Bedfordshire, UK.

The paper considers the problem of joint optimal subcarrier and transmit power allocation with Quality of Service (QoS) guarantee for enhanced packet transmission over Cognitive Radio (CR)-Internet of Vehicles (IoVs) using OFDMA (Orthogonal Frequency Division Multiple Access). A novel wireless radio resource scheduling scheme in OFDMA CR-IoV network systems is proposed. This is proposed for efficient joint transmit power and subcarrier allocation for dynamic spectral resource access in cellular OFDMA-based overlay CRAVNs (Cognitive Radio Assisted Vehicular Networks) in clusters. The objectives of the optimization model applied in this study include the following:

(1) Maximization of the overall system throughput of the CR-IoV system,
(2) Avoiding harmful interference of transmissions of the shared channels’ licensed owners (or primary users),
(3) Guaranteeing the proportional fairness and minimum data-rate requirement of each CR vehicular secondary user (CRV-SU),
(4) Ensuring efficient transmit power allocation amongst CRV-SUs.

Furthermore, a novel approach that uses Lambert-W function characteristics is introduced. Closed-form analytical solutions were obtained in [5] by applying time-sharing variable transformation. Finally, a low-complexity algorithm was developed. This algorithm overcame the iterative processes associated with searching for the optimal solution numerically through iterative programming methods. Theoretical analysis and simulation results demonstrated that under similar conditions, the proposed solutions outperformed the reference scheduler schemes.

The sixth paper [6] is titled “A Deep Learning Approach for Estimating Traffic Density Using Data Obtained from Connected and Autonomous Probes” with authors Daisik Nam, Riju Lavanya, R. Jayakrishnan, Inchul Yang and Woo Hoon Jeon from the University of California at Irvine, USA and the Korea Institute of Civil Engineering and Building Technology, in South Korea.

Traffic density estimation is a challenging problem, since traffic density has a highly nonlinear nature during on-congestion and queue-clearing conditions. To overcome this issue, the focus of this work is the estimation of traffic density from data obtained from Connected and Autonomous Probes (CAPs) and by using LSTM neural networks. The proposed method is designed to learn the input–output relation of Edie’s definition. At the same time, the method recognizes a temporally nonlinear pattern of traffic. The analysis reported in [6] demonstrates that the proposed model accurately estimates traffic density in Free-flow, Transition, and Congested conditions.

The paper [7], titled “Sensor-Aided V2X Beam Tracking for Connected Automated Driving: Distributed Architecture and Processing Algorithms”, with authors from Politecnico di Milano, Italy: M. Brambilla, L. Combi, A. Matera, D. Tagliaferri, M. Nicoli, and U. Spagnolini.

This paper is focused on ultra-reliable low-latency V2X communications able to meet the extreme requirements of high levels of automation. The paper introduces a system architecture and processing algorithms for the alignment of highly collimated V2X beams based either on millimeter-Wave (mmW) or Free-Space Optics (FSO). This work also proposes a V2X architecture that enables a sensor-aided beam-tracking strategy to counteract the detrimental effect of vibrations and tilting dynamics. A parallel low-rate, low-latency, and reliable control link is proposed to be used to exchange data on vehicle kinematics (i.e., position and orientation) that assists the beam pointing along the line-of-sight between V2X transceivers (i.e., the dominant multipath component for mmW, or the direct link for FSO). This link will be based on sub-6 GHz V2X communication, as in the 5G frequency range 1 (FR1). The presented results in the paper show that highly directional mmW and/or FSO communications are promising candidates for massive data-rate vehicular communications, even in high mobility scenarios.

The paper [8], titled “Multi-Camera Vehicle Tracking Using Edge Computing and Low-Power Communication”, is authored by Maciej Nikodem, Mariusz Słabicki, Tomasz Surmacz, Paweł Mrówka, and Cezary Dołęga from three entities established in Poland (Wrocław University of Science and Technology, Polish Academy of Sciences, Neurosoft).

Typical approaches to visual vehicle tracking across a large area require several cameras and complex algorithms to detect, identify, and track the vehicle route. Due to memory requirements, computational complexity, and hardware constrains, the video images are transmitted to a dedicated workstation equipped with powerful graphic processing units. However, this requires large volumes of data to be transmitted and may raise privacy issues. This paper presents a dedicated deep learning detection and tracking algorithms that can be run directly on the camera’s embedded system. This method significantly reduces the stream of data from the cameras, reduces the required communication bandwidth, and expands the range of communication technologies to use. Consequently, it allows the use of short-range radio communication to transmit vehicle-related information directly between the cameras and implement the multi-camera tracking directly in the cameras. The proposed solution includes detection and tracking algorithms as well as a dedicated low-power short-range communication for multi-target multi-camera tracking systems that can be applied in parking and intersection scenarios. System components were evaluated in various scenarios including different environmental and weather conditions.

Paper [9], titled “PortWeather: A Lightweight Onboard Solution for Real-Time Weather Prediction”, is authored by P. Karvelis, D. Mazzei, M. Biviano, C. Stylios, from the University of Ioannina and Computer Technology & Press Diophantus in Greece and from the University of Pisa in Italy.

Meteorology has always had a key role in transportation and mobility systems. Nowadays, the new era of innovative machine learning approaches along with the availability of a wide range of sensors and microcontrollers creates increasing perspectives for providing on-board reliable short-range forecasting of main meteorological variables. The main goal of this study is to propose a lightweight on-board solution for real-time weather prediction. The system is composed of a commercial weather station integrated with an industrial IOT-edge data processing module that computes the wind direction and speed forecasts without the need for an Internet connection. A regression machine learning algorithm was chosen so as to require the smallest amount of resources (memory, CPU) and be able to run in a microcontroller. The algorithm has been designed and coded following specific conditions and specifications. The system has been tested on real weather data gathered from static weather stations and onboard during a test trip. The efficiency of the system has been proven through various error metrics.

Paper [10], titled, “Connected Traffic Data Ontology (CTDO) for Intelligent Urban Traffic Systems Focused on Connected (Semi) Autonomous Vehicles”, is authored by M. Viktorovic, D. Yang, B. De Vriees, from the Technical University of Eindhoven, the Netherlands.

For autonomous vehicles, the ability to share information about their surroundings is crucial. With Level 4 and 5 autonomy in sight, solving the challenge of organization and efficient storing of data, coming from these connected platforms, becomes paramount. Research done up to now has been mostly focused on communication and network layers of V2X data sharing. However, there is a gap when it comes to the data layer. Limited attention has been paid to the ontology development in the automotive domain. More specifically, the way to integrate sensor data and geospatial data efficiently is missing. Therefore, paper [10] proposed developing a new Connected Traffic Data Ontology (CTDO) on the foundations of Sensor, Observation, Sample, and Actuator (SOSA) ontology, to provide a more suitable ontology for large volumes of time-sensitive data coming from multi-sensory platforms, such as connected vehicles. Additionally, as this research aims to further extend the CTDO in the future, a possible way to map to the CTDO with ontologies that represent road infrastructure has been presented. Finally, new CTDO ontology was benchmarked against SOSA, and better memory performance and query execution speeds have been confirmed.

Finally, paper [11], “Research and Implementation of Vehicle Target Detection and Information Recognition Technology Based on NI myRIO”, by authors H. Wang, D. He, J. Yu, and L. Wang, from North University of China, focuses on the problem of vehicle target detection and an information extraction scheme.

In [11], the vehicle information acquisition and processing method based on image recognition is used to design a complete vehicle detection and information extraction system. In the LabVIEW programming environment, the edge detection method is used to realize the vehicle target detection, the pattern matching method is used to realize the vehicle logo recognition, and the Optical Character Recognition (OCR) algorithm is used to realize the vehicle license plate recognition. The feasibility of the design scheme in this paper is verified through the actual test and analysis. The scheme is intuitive and efficient, with high recognition accuracy.

## Data Availability

Not applicable.

## References

[1] Peppa M.V., Komar T., Xiao W., James P., Robson C., Xing J., Barr S. (2021). Towards an end-to-end framework of CCTV-based urban traffic volume detection and prediction. Sensors.

[2] La Manna M., Treccozzi L., Perazzo P., Saponara S., Dini G. (2021). Performance evaluation of attribute-based encryption in automotive embedded platform for secure software over-the-air update. Sensors.

[3] Cheng L., Wang Y., Xue M., Bi Y. (2020). An indoor robust localization algorithm based on data association technique. Sensors.

[4] Stuparu D.G., Ciobanu R.-I., Dobre C. (2020). Vehicle detection in overhead satellite images using a one-stage object detection model. Sensors.

[5] Eze J., Eze E., Zhang S., Liu E.L. (2020). Design optimization of resource allocation in OFDMA-based cognitive radio-enabled Internet of Vehicles (IoVs). Sensors.

[6] Nam D., Lavanya R., Jayakrishnan R., Yang I., Jeon W.H. (2020). A deep learning approach for estimating traffic density using data obtained from connected and autonomous probes. Sensors.

[7] Brambilla M., Combi L., Matera A., Tagliaferri D., Nicoli M., Spagnolini U. (2020). Sensor-aided V2X beam tracking for connected automated driving: Distributed architecture and processing algorithms. Sensors.

[8] Nikodem M., Słabicki M., Surmacz T., Mrówka P., Dołęga C. (2020). Multi-camera vehicle tracking using edge computing and low-power communication. Sensors.

[9] Karvelis P., Mazzei D., Biviano M., Stylios C. (2020). PortWeather: A lightweight onboard solution for real-time weather prediction. Sensors.

[10] Viktorovic M. (2020). Connected Traffic Data Ontology (CTDO) for intelligent urban traffic systems focused on connected (Semi) autonomous vehicles. Sensors.

[11] Wang H., He D., Yu J., Wang L. (2020). Research and implementation of vehicle target detection and information recognition technology based on NI myRIO. Sensors.

